# Mindfulness-Based Cancer Recovery (MBCR) versus Supportive Expressive Group Therapy (SET) for distressed breast cancer survivors: evaluating mindfulness and social support as mediators

**DOI:** 10.1007/s10865-016-9799-6

**Published:** 2016-10-08

**Authors:** Melanie. P. J. Schellekens, Rie Tamagawa, Laura E. Labelle, Michael Speca, Joanne Stephen, Elaine Drysdale, Sarah Sample, Barbara Pickering, Dale Dirkse, Linette Lawlor Savage, Linda E. Carlson

**Affiliations:** 1 Radboud Centre for Mindfulness, Department of Psychiatry, Radboud University Medical Centre, Reinier Postlaan 4, P.O. Box 9101, 6500 HB Nijmegen, The Netherlands; 2 Department of Oncology, University of Calgary, Calgary, AB Canada; 3 Department of Psychosocial Oncology, Tom Baker Cancer Centre, Calgary, AB Canada; 4 British Columbia Cancer Agency, Vancouver, BC Canada; 5 Department of Psychiatry, University of British Columbia, Vancouver, BC Canada

**Keywords:** Mindfulness-Based Cancer Recovery, Mindfulness, Supportive Expressive Group Therapy, Social support, Mediation, Breast cancer

## Abstract

Despite growing evidence in support of mindfulness as an underlying mechanism of mindfulness-based interventions (MBIs), it has been suggested that nonspecific therapeutic factors, such as the experience of social support, may contribute to the positive effects of MBIs. In the present study, we examined whether change in mindfulness and/or social support mediated the effect of Mindfulness-Based Cancer Recovery (MBCR) compared to another active intervention (i.e. Supportive Expressive Group Therapy (SET)), on change in mood disturbance, stress symptoms and quality of life. A secondary analysis was conducted of a multi-site randomized clinical trial investigating the impacts of MBCR and SET on distressed breast cancer survivors (MINDSET). We applied the causal steps approach with bootstrapping to test mediation, using pre- and post-intervention questionnaire data of the participants who were randomised to MBCR (*n* = 69) or SET (*n* = 70). MBCR participants improved significantly more on mood disturbance, stress symptoms and social support, but not on quality of life or mindfulness, compared to SET participants. Increased social support partially mediated the impact of MBCR versus SET on mood disturbance and stress symptoms. Because no group differences on mindfulness and quality of life were observed, no mediation analyses were performed on these variables. Findings showed that increased social support was related to more improvement in mood and stress after MBCR compared to support groups, whereas changes in mindfulness were not. This suggests a more important role for social support in enhancing outcomes in MBCR than previously thought.

## Introduction

Mindfulness-Based Stress Reduction (MBSR) (Kabat-Zinn, 1990) and Supportive Expressive Group Therapy (SET) (Classen et al., 1993) are two frequently studied and well-validated psychosocial group interventions for cancer patients. The MBSR program was adapted by us for the treatment of cancer patients and is called Mindfulness-Based Cancer Recovery (MBCR) (Carlson & Speca, 2010). Several clinical trials on MBCR and other mindfulness-based interventions (MBIs) have shown efficacy in improving psychosocial outcomes in cancer patients, such as mood disturbance, stress symptoms and quality of life (e.g., Carlson et al., 2003; Johannsen et al., 2016; Lengacher et al., 2016; Speca et al., 2000; Wurtzen et al., 2013). Recent meta-analyses provide further support for the effectiveness of MBIs in cancer with moderate to large effect sizes (Cramer et al., 2012; Piet et al., 2012; Zhang et al., 2016). While SET differs from MBCR in terms of content, focus and theoretical underpinnings, the programs are similar regarding group format, group size and contact hours. In several clinical trials, SET also demonstrated its effectiveness in reducing stress symptoms and improving quality of life (e.g., Classen et al., 2001; Kissane et al., 2007).

Gaining a deeper understanding of *how* interventions work can advance treatment research (Kazdin, 2007; Moyer et al., 2012). Mediation analyses may help identify the active components of the intervention and subsequently the intervention can be optimized by tailoring the program accordingly (Kazdin, 2007). Studies examining the mechanisms of an intervention typically focus on the theoretical foundation of the program. SET is based on the idea that participants learn to better cope with their cancer and feel less distressed by expressing emotions and increasing the experience of social support. Clinical trials have examined the effects of SET on potential working mechanisms, finding a decrease in suppression of negative affect and improvements in social functioning (Giese-Davis et al., 2002; Kissane et al., 2007). However, to the extent of our knowledge, no study has examined whether changes in these potential working mechanisms mediated the effects of SET.

In MBIs, participants learn to develop non-judgmental and accepting awareness of experiences by practicing mindfulness, which in turn results in a decrease of psychological distress (Kabat-Zinn, 1990; Segal et al., 2002). A recent meta-analysis of 12 studies concluded that there is consistent moderate evidence for increases in mindfulness as a mechanism of MBIs (Gu et al., 2015). This mediation process, however, has mainly been studied in comparison with waitlist and usual care control groups. Such research designs cannot rule out whether non-specific factors such as social support may also underlie the positive effects of MBIs (Chiesa, 2011). Independent of the content of an intervention, being in a group with fellow patients and sharing personal experiences can create feelings of social support. In turn, the frequency and quality of social networks has often been positively associated with wellbeing and health (Reblin and Uchino, 2008), potentially explaining the intervention effects. A recent meta-ethnography based on 14 qualitative studies indicated that social support can play an important role in MBIs (Malpass et al., 2012). Through the practice of non-judgemental awareness, MBI can provide an atmosphere that fosters the allowing and accepting of thoughts and emotions, facilitating openness and sharing experiences with one another (Schellekens et al., 2016). Such a supportive environment can help patients to facilitate each other’s learning processes (Mackenzie et al., 2007; Schellekens et al., 2016).

The aim of the present study was to examine whether change in mindfulness and/or social support mediated the effect of MBCR compared to SET, on change in mood disturbance, stress symptoms and quality of life among distressed breast cancer survivors. As MBCR is mainly focused on practicing mindfulness and SET on facilitating support, we hypothesized that enhancement in mindfulness would mediate the effects of MBCR while enhancement in social support would mediate the effects of SET.

## Method

### Participants

This mediation study was embedded in a multi-site randomized clinical trial investigating the impacts of MBCR and SET on distressed breast cancer survivors in Calgary and Vancouver (the MINDSET trial, NCT00390169) (Carlson et al., 2013). A total of 271 women with a diagnosis of stage I, II, or III breast cancer with significant distress were randomized into either MBCR, SET or a minimal-treatment waitlist control group (ratio 2:2:1). The protocol was approved by the institutional review board at both centres. The eligibility criteria, recruitment approach and CONSORT diagram are described fully in the main outcome paper (Carlson et al., 2013). The current study used the pre- (Time 1) and post-intervention (Time 2) questionnaire data of survivors who were randomized to MBCR or SET. The 12-month follow-up data was also considered for mediation analysis. However, as a large number of participants failed to complete the 12-month follow-up data, the remaining sample size was rather small (n_MBCR_ = 51; n_SET_ = 55) and we decided to focus on post-intervention data for the mediation analysis (n_MBCR_ = 69; n_SET_ = 70).

### Interventions

#### MBCR

The intervention was modelled on the MBSR program originally developed at the Massachusetts Medical Center (Kabat-Zinn, 1990). The program was modified as MBCR to make it more suitable for cancer patients (Carlson & Speca, 2010), and a number of studies have validated its efficacy in this context (Carlson et al., 2013; Carlson et al., 2007; Carlson et al., 2004; Garland et al., 2014; Speca et al., 2000). At each class, participants engaged in mindfulness practice consisting of gentle yoga and meditation exercises. The instructors provided information about mind–body interactions and led discussions on mindfulness practice. The program consisted of 8 weekly group sessions of 90 min each plus a 6-h silent retreat between weeks 6 and 7 for a total of 18 contact hours. Sessions were led by trained instructors who have facilitated previous MBCR trials. Participants were integrated into regularly offered MBCR groups of up to 20 participants with a variety of cancer types.

#### SET

The SET group was based on the manualized treatment developed by the Psychosocial Treatment Laboratory’s Breast Cancer Intervention Program at Stanford University (Classen et al., 1993). The goals of the therapy include facilitating mutual and family support, enhancing openness and emotional expressiveness, integrating a changed self and body image into the view of self, improving coping skills and doctor-patient relationships, and detoxifying feelings around death and dying. The program consisted of 12 weekly group sessions of 90 min each. The therapists in the current study were experienced in facilitating multisite trials on SET. As with MBCR, participants were integrated into regularly offered clinical groups of up to 12 participants.

## Measures

Demographics (age, education, employment, marital status) were assessed. Disease characteristics (cancer stage, date of diagnosis) were determined based on chart reviews.

### Outcome measures

#### Mood disturbance

The six subscales of the Profile of Mood States (POMS) (anxiety, depression, anger, vigor, fatigue, and confusion) (McNair et al., 1971) were summed to form a total mood disturbance score. The POMS has been widely used in psychiatric and medical populations, including patients with cancer (Cassileth et al., 1986).

#### Stress

The 56-item Calgary Symptoms of Stress Inventory (CSOSI) measures physical, psychological and behavioural responses to stressful situations via 8 subscales (Carlson and Thomas, 2007). The total score was used.

#### Quality of life

The Functional Assessment of Cancer Therapy–Breast (FACT-B) (Brady et al., 1997) is designed to measure several dimensions of quality of life, via a general cancer quality of life measure and a breast cancer subscale with items specific to quality of life in breast cancer patients. The total scale was used.

### Potential mediators

#### Social support

The 19-item Medical Outcomes Study Social Support Survey (MOS-SSS) yields scores on four dimensions (emotional or informational support, tangible support, affectionate support and positive social interaction), which were summed to form a total functional social support score (Sherbourne and Stewart, 1991).

#### Mindfulness

The 15-item Mindful Attention Awareness Scale (MAAS) (Brown and Ryan, 2003) is designed to measure present-centered attention and awareness. It has been validated in general populations (Brown and Ryan, 2003) as well as in cancer patients (Carlson and Brown, 2005).

### Statistical analysis

All analyses were conducted using (1) a study completer sample (those who completed both pre- and post-questionnaires) and (2) an intervention completer sample (those who completed both questionnaires and also received a significant dose of the intervention, i.e. at least half of the program sessions: > 4 MBCR sessions or > 5 SET sessions).

#### Effect of group participation

To examine the effect of MBCR and SET on mood disturbance, stress, quality of life, mindfulness and social support, ANCOVAs were conducted with condition (MBCR versus SET) as the independent variable, Time 2 scores as the dependent variable, and Time 1 scores as the covariate. Cohen’s d effect size was calculated based on the mean group difference between MBCR and SET adjusted for baseline scores, divided by the pooled baseline standard deviation. The following formula was used, Cohen’s $d = \left( {\Delta M_{\text{MBCR}} - \Delta M_{\text{SET}} } \right) / \sigma_{{{\text{pooled}}T1 }}$ while $\sigma_{{{\text{pooled}}T1 }} = \sqrt {\left( {\sigma_{{{\text{MBCR}}T1 }}^{2} + \sigma_{{{\text{SET}}T1 }}^{2} } \right)/ 2}$ (Cohen, 1988).

#### Mediation analyses

Separate mediation analyses were conducted to determine whether improvements in mood disturbance, stress, and quality of life after MBCR versus SET participation were mediated by: (1) mindfulness and (2) social support. First, residualized change scores (Time 2 predicted by Time 1 scores) were computed for the POMS, CSOSI, FACT-B, MAAS and MOS-SSS. Second, the causal steps mediation approach was applied (Baron & Kenny, 1986). This regression-based mediation model assumes that the independent variable is associated with changes in the mediator, which is in turn associated with changes in the outcome. Mediation is confirmed when the direct effect of the independent variable on outcome decreases with the inclusion of the mediator (Baron & Kenny, 1986).

Third, following recommendations for examining mediation (MacKinnon et al., 2002; MacKinnon et al., 2004), a nonparametric bootstrapping procedure for testing the statistical significance of the indirect (or mediated) effect was applied (Preacher & Hayes, 2008; Shrout & Bolger, 2002). This method does not require that the sampling distribution of the indirect effect is symmetrical or normal and it offers more power relative to traditional approaches while maintaining efficient control over the Type I error rate (Fritz & MacKinnon, 2007; MacKinnon et al., 2002; Preacher & Hayes, 2008). Preacher and Hayes’ (2004) SPSS bootstrapping script was used to derive bias-corrected and accelerated 95 % confidence intervals by taking 5000 random samples for the indirect effect of group (MBCR versus SET) through the hypothesized change in the mediator on change in outcome scores. Mediation is said to occur if the derived confidence intervals does not contain zero (Preacher and Hayes, 2004).

## Results

### Participants

Of the 113 breast cancer survivors randomized to MBCR, 44 failed to complete pre- and post-questionnaires, leaving 69 participants that completed both questionnaires. This is the group we call ‘study completers’. On average they attended 6.8 out of 9 MBCR sessions (SD = 2.0), of which 71 % participated in 7 sessions or more. Of the study completers, 15 women dropped out MBCR, leaving 54 participants that completed the questionnaires *and* the intervention. This is the group we call ‘intervention completers’. Of the 104 participants randomized to SET, 34 did not complete the questionnaires, leaving 70 participants completing both questionnaires (i.e. study completers). On average they attended 9.3 out of 12 SET sessions (SD = 2.9), of which 76 % participated in 9 sessions or more. Of these study completers, 9 dropped out SET, leaving 60 participants that completed the questionnaires *and* the intervention (i.e. intervention completers).

In both groups, the main reasons for dropping out the intervention and the study were scheduling conflict (n = 4) or illness progression (n = 3). In addition, several participants could not be reached for post-assessment (n = 10). The vast majority of participants, however, did not provide a reason for dropping out. Baseline characteristics of participants completing both questionnaires are shown in Table 1. *T* tests and Chi square tests did not reveal baseline differences between the MBCR and SET group on any variable (all *p* values > .05).

**Table 1:** Demographic and clinical characteristics of 139 participants

	MBCR	(*n* = 69)	SET	(*n* = 70)
	*M*	*(SD)*	*M*	*(SD)*
Age (years)	54.9	(9.2)	53.2	(9.8)
Education (years)	15.7	(3.1)	15.7	(3.0)
Employment, *n* (%)				
Unemployed/retired/disabled	24	(34.8)	28	(40.0)
Part time	18	(26.1)	15	(21.4)
Full time	26	(37.7)	25	(35.7)
Marital status, *n* (%)				
Single	9	(13.0)	15	(21.4)
Cohabiting/married	43	(62.3)	42	(60.0)
Divorced/separated/widowed	14	(20.3)	11	(15.7)
Months since diagnosis	24.5	(18.0)	23.3	(18.4)
Cancer Stage, *n* (%)				
0	2	(2.9)	1	(1.4)
1	28	(40.6)	35	(50.0)
2	26	(37.7)	20	(28.6)
3	6	(8.7)	9	(12.9)
4	0	(0.0)	1	(1.4)

### Effect of MBCR versus SET participation

#### Study completers

Three separate ANCOVAs revealed that mood disturbance (*F*(1, 136) = 8.64, *p* = .004) and stress symptoms (*F*(1, 136) = 9.40, *p* = .003) were significantly decreased at Time 2 in MBCR compared to SET, while no significant effect of group was observed for quality of life (*F*(1, 134) = 3.10, *p* = .080). As there was no effect on quality of life that could be mediated, no further mediation analysis was performed for this variable. In addition, social support (*F*(1, 136) = 4.29, *p* = .040) but not mindfulness (*F*(1, 124) = .026, *p* = .872) increased significantly from pre- to post-intervention in MBCR versus SET. Mindfulness did not meet the criteria of a mediator, so no further analyses were performed for this variable. Table 2 displays the effects of MBCR versus SET on the outcome and mediator variables.

**Table 2:** Effect of MBCR (n = 69) versus SET (n = 70) on study variables

	Time 1		Time 2		*p* ^A^	Cohen’s *d*
	*M*	(*SD*)	*M*	(*SD*)		
Mood disturbance (POMS)						
MBCR	33.62	(31.86)	14.48	(26.72)	.004	.34
SET	36.30	(37.47)	28.85	(39.14)		
Stress symptoms (CSOSI)						
MBCR	67.98	(28.21)	48.77	(27.83)	.003	.35
SET	69.26	(33.67)	61.04	(33.50)		
Quality of Life (FACT-B)						
MBCR	95.43	(21.60)	107.36	(18.80)	.080	.22
SET	97.09	(23.54)	103.34	(22.35)		
Mindfulness (MAAS)^B^						
MBCR	3.81	(0.86)	4.16	(0.98)	.872	.28
SET	3.88	(0.84)	4.05	(0.79)		
Social Support (MOS-SSS)						
MBCR	65.14	(22.02)	69.54	(21.09)	.040	.37
SET	70.61	(19.79)	68.73	(21.40)		

^A^ANCOVA predicting MBCR versus SET on Time 2 scores, controlling for Time 1 scores

^B^Data is missing from 15 patients in the MBCR group and 14 patients in the SET group

#### Intervention completers

Mood disturbance (*F*(1, 121) = 7.82, *p* = .006), stress symptoms (*F*(1, 121) = 8.68, *p* = .004) and quality of life (*F*(1, 119) = 4.07, *p* = .046) significantly improved after MBCR versus SET. Regarding the potential mediators, no significant effect of group was found for social support (*F*(1, 121) = 2.70, *p* = .103) or mindfulness (*F*(1, 96) = 1.62, *p* = .207). As both potential mediators did not meet the criteria of a mediator, no further mediation analyses were performed in the sample of intervention completers.

### Social support as mediator of MBCR versus SET participation

#### Mood disturbance

**Fig. 1:**
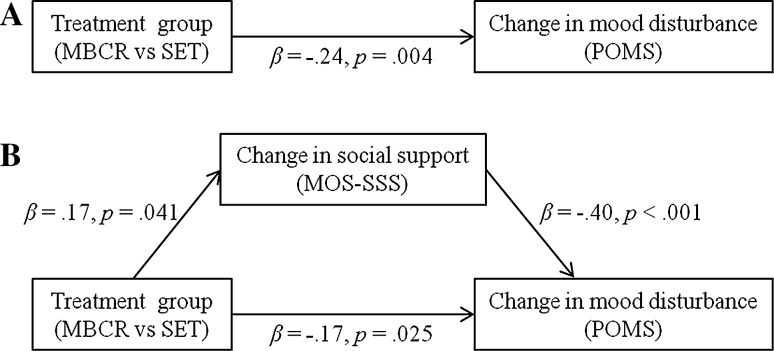
Path diagrams representing **a** the direct effect of treatment group on change in mood disturbance and **b** the mediation model with the indirect effect of treatment group via change in social support on change in mood disturbance, decreasing the direct effect of treatment group on change in mood disturbance, indicating partial mediation

Regression analyses revealed a significant effect of treatment group on changes in mood disturbance (β = −.24, *p* = .004), ascertaining that there was an effect to be mediated (Fig. 1a). Treatment group showed a significant effect on change scores for social support (β = .17, *p* = .041) (Fig. 1b), and the increase in social support significantly predicted change in mood disturbance (β = −.40, *p* < .001). Including change in social support in the model, the effect of treatment group on mood disturbance was reduced; the *p* value increased but remained significant (β = −.17, *p* = .025), indicating that social support partially mediated the effect of MBCR versus SET on improvement in mood. The bootstrap analysis indicated that the mediated effect was statistically significant (95 % CI did not contain zero: −.312 and −.016).

#### Stress symptoms

**Fig. 2:**
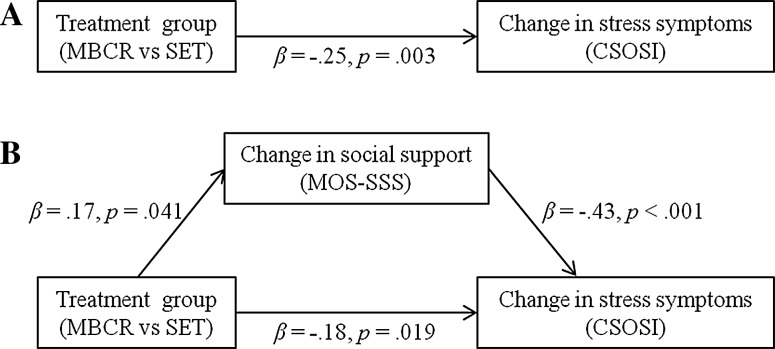
Path diagrams representing **a** the direct effect of treatment group on change in stress symptoms and **b** the mediation model with the indirect effect of treatment group via change in social support on change in stress symptoms, decreasing the direct effect of treatment group on change in stress symptoms, indicating partial mediation

Treatment group had a significant effect on the change in stress symptom scores (β = −.25, *p* = .003) (Fig. 2a). As indicated above, treatment group predicted changes in social support (β = .17, *p* = .041). In turn, the increase in social support significantly predicted changes in stress symptoms (β = −.43, *p* < .001) (Fig. 2b). With change in social support included as a mediator, the effect of treatment group was reduced; the p-value increased but remained significant (β = −.18, *p* = .019). These results indicate that change in social support also partially mediated the effect of MBCR versus SET on change in stress symptoms. The bootstrap analysis indicated that the mediated effect was statistically significant (95 % CI −.321 and −.014).

## Discussion

The goal of the present research was to examine potential mediators underlying the effect of MBCR and SET on psychological outcomes of breast cancer survivors within a randomized clinical trial. We expected changes in mindfulness to be related to participation in MBCR and social support to be more strongly affected in SET. Surprisingly, social support increased more after MBCR than after SET, and this change partially mediated the effect of MBCR on mood and stress symptoms. While fostering group support in MBCR is more an implicit part of the program than in SET, where it is a central objective, MBCR seems to provide an environment where breast cancer survivors support and feel supported by one another. These findings are in line with qualitative studies (Malpass et al., 2012), showing how MBIs provide a safe environment, which enhances a sense of community (Schellekens et al., 2016) and facilitates patients to learn from one another (Mackenzie et al., 2007; van den Hurk et al., 2015). MBCR also includes content and experiential practice focused on the cultivation of loving-kindness. Learning to direct kind and compassionate attention towards oneself and others may enhance feelings of relatedness and support within and outside of the group (Birnie et al., 2010). Note that in the subsample of women who completed both the questionnaires as well as the intervention the effect on social support disappeared. A possible explanation could be the drop in sample size from 139 to 125 women, which limits the power and increases the chance of a type II error occurring.

Thus far, mediation studies on MBIs have focused on working mechanisms within the individual (Gu et al., 2015) whereas mediators on the level of the group such as social support, seem to potentially be of equal importance. The mediating effect of social support could be seen as a non-therapeutic outcome inherent to a group-based intervention (Chiesa, 2011). However, by comparing MBCR with another group-based training, the increase in social support appears to be an underlying mechanism that might be unique to MBCR and partly explains its positive effects in cancer patients. Note that social support only partly explains the effect of group on mood disturbance and stress symptoms. Other working mechanisms of MBCR affecting outcomes were not addressed in this analysis, but may also be important. These may include the promotion of self-compassion and emotion regulation skills (for an overview, see Gu et al., 2015).

Surprisingly, no effect was found on mindfulness after MBCR versus SET. This finding is in contrast with several studies showing that the change in mindfulness after MBI participation mediates the effects on several outcomes (Gu et al., 2015). A possible explanation for these contrasting results might be the use of the MAAS, as it only measures one aspect of mindfulness, i.e. the presence/absence of attention to and awareness of present moment experiences during daily activities, or put differently, the opposite of “running on automatic pilot”. Possibly, SET participants may also become more aware of their emotions, thoughts and behaviours during daily experiences because of the programme’s emphasis on expressing emotions and improving coping skills. Other instruments, such as the Five Facet Mindfulness Questionnaire (FFMQ) (Baer et al., 2006), might measure mindfulness skills that are more specifically practiced in MBCR than in SET, e.g. Observing and Nonjudging of inner experiences. In our previous work with the FFMQ, MBCR had the strongest effect on the Observing facet compared to other FFMQ facets and MAAS (Labelle et al., 2014). Baer and colleagues also demonstrated that the Observing facet appears to be particularly sensitive to meditation experience (Baer et al., 2006, 2008).

A number of limitations should be noted. The study sample consisted solely of women with breast cancer, of which the majority was highly educated and on average they received their cancer diagnosis 2 years prior to participation, limiting the generalizability of these findings. To date, the vast majority of study participants in MBIs for cancer patients have been women with breast cancer (Piet et al., 2012). Future research should also examine the effectiveness and potential working mechanisms of MBIs in other types of cancer. Another limitation is the relatively high intervention drop-out rates. It might bias the sample and decrease generalizability of the results. As most participants did not provide a reason for dropping out, we do not know whether attrition is related to practical reasons, such as scheduling conflicts, or due to the high level of burden data collection presented to them. In addition, the sample of follow-up data was limited, preventing us from including it in the mediation analysis. Consequently, both the mediator as well as the outcome variables were assessed before and after the intervention, which limits conclusions about what changed first, social support or mood/stress symptoms. Future studies should take the temporal order of the mediator and outcome variable into account, exploring whether early changes in the mediator predict later changes in the outcome (Labelle et al., 2014). In previous work, for example, we demonstrated that early increase in observing (i.e., change during first half of MBCR) predicted later increase in awareness of daily activities (i.e., change during second half of MBCR) (Labelle et al., 2014). Another limitation is that we relied on a self-report questionnaire for assessing mindfulness measuring only one facet of mindfulness (i.e. attention to and awareness of daily experiences). While the validity of the measure is under debate (Grossman & Van Dam, 2011), the scale has high internal consistency and has been adopted successfully in studies on the effects of mindfulness (Brown & Ryan, 2003), and for use in people with cancer (Carlson & Brown, 2005). In addition, the MAAS was filled out by a smaller sample of participants (n = 110) than the other questionnaires (n = 139) due to procedural changes in the protocol partway through the study, potentially decreasing statistical power to detect group differences.

The present study implies that the group-based character of MBCR is of added value to breast cancer survivors’ mood and stress. This implication should be seen in the light of an increase in MBIs that are adapted to the individual in both clinical practice as well as in research settings (Compen et al., 2015; Tovote et al., 2014; Wahbeh et al., 2014; Schroevers et al., 2016). For patients who are unwilling or unable to participate in a group due to disabilities or constrained time schedules, an individual MBI program appears to be a good solution. However, these patients will miss out on the group support and observational learning that typically occurs in group-based settings. Future non-inferiority trials should examine whether individual-based and group-based MBIs are equally effective in improving mood and stress in cancer patients. In addition, it would be interesting to examine whether the central role of social support only holds for (breast) cancer or also generalizes to other MBI target groups. When the main motivation for participants is learning to cope with day-to-day stressors rather than coping with a life-threatening diagnosis, actual mindfulness practice might be more important than social support. Testing social support as a mediator across populations and MBIs will inform our understanding of this intervention and may lead to program modifications that might maximize the effectiveness of MBIs.
